# Understanding drivers of wild oyster population persistence

**DOI:** 10.1038/s41598-021-87418-1

**Published:** 2021-04-09

**Authors:** Mickael Teixeira Alves, Nick G. H. Taylor, Hannah J. Tidbury

**Affiliations:** Centre for Environment, Fisheries and Aquaculture Science, International Centre of Excellence for Aquatic Animal Health, Weymouth, DT4 8UB UK

**Keywords:** Climate-change ecology, Ecological modelling, Ecosystem ecology, Population dynamics

## Abstract

Persistence of wild Pacific oyster, *Magallana gigas*, also known as *Crassostrea gigas*, has been increasingly reported across Northern European waters in recent years. While reproduction is inhibited by cold waters, recent warm summer temperature has increased the frequency of spawning events. Although correlation between the increasing abundance of Pacific oyster reefs in Northern European waters and climate change is documented, persistence of wild populations may also be influenced by external recruitment from farmed populations and other wild oyster populations, as well as on competition for resources with aquaculture sites. Our understanding of the combined impact of the spawning frequency, external recruitment, and competition on wild population persistence is limited. This study applied an age-structured model, based on ordinary differential equations, to describe an oyster population under discrete temperature-related dynamics. The impact of more frequent spawning events, external recruitment, and changes in carrying capacity on Pacific oyster density were simulated and compared under theoretical scenarios and two case studies in Southern England. Results indicate that long term persistence of wild oyster populations towards carrying capacity requires a high frequency of spawning events but that in the absence of spawning, external recruitment from farmed populations and other wild oyster populations may act to prevent extinction and increase population density. However, external recruitment sources may be in competition with the wild population so that external recruitment is associated with a reduction in wild population density. The implications of model results are discussed in the context of wild oyster population management.

## Introduction

The Pacific oyster, *Magallana gigas* (also known as *Crassostrea gigas*^1^) is native to warm temperate regions, specifically the Northwest Pacific and Sea of Japan^2^. The species was intentionally introduced, outside of its native range, into European waters in the mid-1960s for commercial purpose in response to the decline of native oyster stocks^3^. It has since become one of the most cultivated shellfish in Northern Europe. In the UK, annual production is circa 1200 tonnes which has a direct sale value close to £5 million^4^. Impacts of the Pacific oyster outside of its native range, including in temperate regions, have been documented, and encompass ecological impacts, such as displacement of native species and habitat and socio-economic impacts, such as injuries caused by shells on leisure beaches^5,6^. This species is therefore widely considered as an invasive non-native species (INNS)^7^. While its risk has been historically categorized between “medium” and “high” with respect to UK waters, its recent classification in England is as an “ordinarily resident medium risk INNS”^8^. Management of wild populations and the use of this species in aquaculture are therefore emotive policy subjects, requiring consideration of both the value of this species alongside its potential impacts.

Temperature is a critical factor for the Pacific oyster life cycle and strongly influences the establishment of wild populations^9^. In a temperate climate, *M. gigas* has a seasonal reproductive cycle with maturity and spawning occurring in summer, at temperatures between 18 and 20 $^\circ \hbox {C}$^2,10,11^, though partial and complete spawning below 18 $^\circ \hbox {C}$ has been documented^12–15^. *M. gigas* has an r-type reproduction that involves a high reproductive capacity with 50 to 200 million eggs produced per individual during spawning^16^ but also a larval mortality as large as 99%^17^. Fertilization occurs in the water column, which if successful results in development of planktonic larvae which then metamorphose into juvenile spat^18^. Larval development and settlement are temperature-dependent and can take from 2 to 4 weeks at 25 $^\circ \hbox {C}$ and 17 $^\circ \hbox {C}$, respectively^14,15,19,20^, with a longer duration increasing risk of exposure to predators^21^. Juvenile spat, once settled, develop into adults in 1–3 years^22^ but are sensitive to low temperatures and cannot survive below 3 $^\circ \hbox {C}$^23^. Wild adult oysters can live 20 to 40 years and are known to tolerate wide temperature ranges^24^. Historic temperatures in North West Europe allowed growth and cultivation but inhibited reproduction in the northernmost regions, thereby preventing successful wild establishment^12,25^.

Established and self-sustaining wild oyster reefs have been reported in French, Dutch, German, Danish Wadden and Scandinavian waters, including in areas thought too cold for larval recruitment^5,12,26^. Sporadic settlements of Pacific oysters were detected in the early-1990s in the UK^27^. Likely the consequence of global climate change, persistent wild *M. gigas* populations are now reported throughout the UK coastline, with substantial settlements in the South of England and Wales, and more isolated individual settlement occasionally seen in more northern and cooler waters^28,29^. In particular, warmer summer temperature has increased the frequency of spawning events and facilitated development and maturation of *M. gigas*, therefore aiding settlement and persistence of populations in the UK^11,28,30–33^, and other parts of the North West Europe^10,12^.

While a positive correlation between the presence of Pacific oyster reefs in North West Europe and temperature increases associated with climate change is documented, persistence of wild oysters can be unpredictable even under favorable temperature conditions^11,25^. In addition to the influence of temperature on spawning, wild populations may experience external recruitment from farmed populations and other wild oyster populations^34^. Competition for resources between farmed oyster populations and native filter feeders has been evidenced and may occur if farming is intensive enough to impact the carrying capacity of the local environment^35–38^. Despite being integral to predicting changes in Pacific oyster distribution and associated impact, our understanding of the potentially complex interactions between spawning frequency, external recruitment, and competition for resource in relation to wild population persistence is limited.

By developing and implementing a stage-structured Pacific oyster population model based on a series of ordinary differential equations inspired by *Crassostrea virginica* habitat models^39,40^, this study aims to increase our understanding of the occurrence and persistence of wild populations under temperature-dependent spawning and external recruitment from farmed or wild populations. Specifically, the model was applied with the aim of helping to understand the potential influence of increased temperature driven spawning events and external recruitment from aquaculture and remote wild populations on the dynamics of wild oyster populations under different carrying capacity scenarios. It is hoped that this study will contribute evidence to inform future management decisions.

## Results

### Theoretical scenarios

#### Changes in spawning frequency

**Figure 1:**
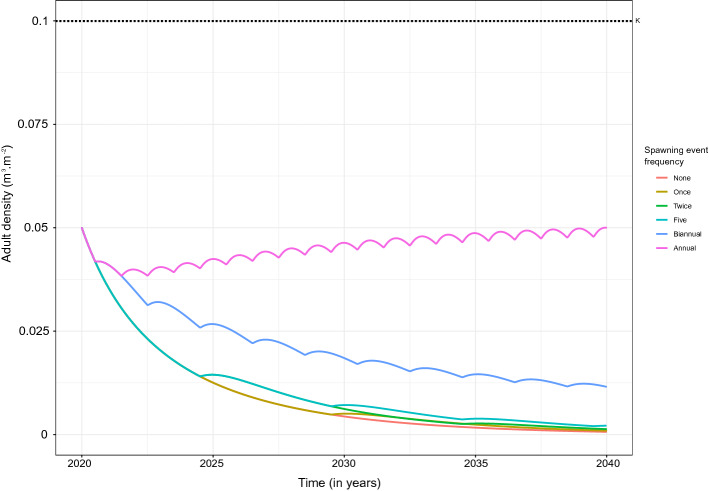
Adult density of the wild population ($\hbox {m}^3\,\hbox {m}^{-2}$) over time with no spawning event (*None*—orange line), one spawning event after 10 years (*Once*—ocher line), two spawning events after 5 and 15 years (*Twice*—green line), spawning events every 5 years (*Five*—clear blue line), biannual spawning events (*Biannual*—purple line), and annual spawning events (*Annual*—pink line). The wild population is assumed to be initially established at a density $A_0=0.05\,\hbox {m}^3\,\hbox {m}^{-2}$ and the carrying capacity at $K=0.1\,\hbox {m}^3\,\hbox {m}^{-2}$ (black dotted line).

Model outputs suggest that Pacific oyster adult population density is strongly dependent on the frequency of spawning events, i.e. the frequency with which the spawning threshold temperature, set at 19.7 $^\circ \hbox {C}$, is reached (Fig. 1). In the absence of external recruitment sources, an already established population can persist at low levels as a result of the long lifespan of Pacific oyster. However, long-term increase in adult density of an established population requires the spawning threshold temperature to be reached every year. Following an initial decline in density, reflecting the period until juveniles settle, annual within population spawning acts to continuously increase the adult population density. After 20 years the density reaches 50% of its maximum carrying capacity set at $0.1\,\hbox {m}^3\,\hbox {per m}^{2}$. Annual oscillations in adult population density occur as a result of seasonal recruitment of larvae following an annual within population spawning event.

If the spawning threshold temperature is not reached every year, adult population density decreases in the long term as mortality exceeds natural recruitment. Under biannual within population spawning frequency, a 20-year adult density decreases to 10% of its maximum carrying capacity, which corresponds to an 80% reduction in density compared to under annual spawning frequency. Simulations showed that adult population density is slightly higher after 20 years when within population spawning occurs every 5 years compared to not at all. The adult density increases as a result of each within population spawning event, which is illustrated by a distinct step-wise increase in adult population density every 5 years under a 5 year within population spawning event scenario. In the absence of within population spawning or when within population spawning frequency is less than or equal to once every 5 years, the adult population decreases substantially, nearing extinction within 20 years which is in line with the life-span of Pacific oyster.

#### Changes in external recruitment levels

**Figure 2:**
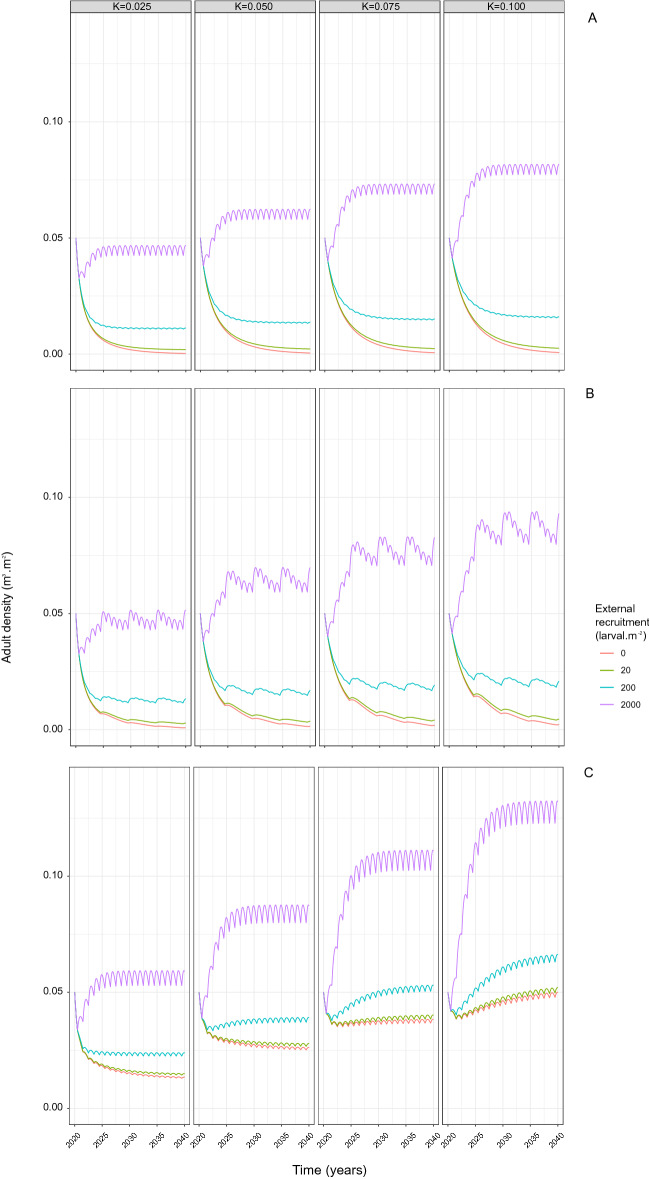
Adult density of the wild population ($\hbox {m}^3\,\hbox {m}^{-2}$) over time with (**A**) no spawning event, (**B**) spawning events every 5 years, and (**C**) annual spawning events, for 4 levels of external recruitment (orange—R = 0 $\hbox {larvae m}^{-2}$, green—R = 20 $\hbox {larvae m}^{-2}$, blue—R = 200 $\hbox {larvae m}^{-2}$, purple—R = 2000 $\hbox {larvae m}^{-2}$) and 4 carrying capacities (from the left to the right, $K=\{0.025; 0.05; 0.075; 0.1\}$. The wild population is assumed to be initially established at a density $A_0=0.05\,\hbox {m}^3\,\hbox {m}^{-2}$.

In the presence of annual external recruitment, reflecting influx of larvae from farmed and other wild populations under favorable environmental conditions, the adult population density is predicted to be higher, under all scenarios, than in the absence of external recruitment (Fig. 2). In the absence of within population spawning, low level of external recruitment prevents the population becoming extinct after 20 years (Fig. 2A). Increased within population spawning frequency is predicted to further increase adult population density under all external recruitment levels, which is illustrated with population spawning frequency equal to once every 5 years (Fig. 2B) and annually (Fig. 2C). However, the interaction between external recruitment and within population spawning frequency suggests that a wild population which spawns annually but has no external recruitment can reach higher adult densities at year 20 than a population that spawns less frequently but has low to medium external recruitment.

The relative increase in predicted density is positively associated with the level of external recruitment, in addition to the frequency of within population spawning events. As each external recruitment event fuels the adult population through influx of larvae and subsequently juveniles, the population density exceeds the carrying capacity which, through density-dependent growth, is subject to a decreasing trend between each spawning event, irrespective of the level of external recruitment and carrying capacity. Similarly, under all within population spawning frequencies and high external recruitment (R = 2000 larvae per $\hbox {m}^2$), the adult density exceeds the carrying capacity with periodic influx of larvae through recruitment maintaining it at high levels.

#### Reduction in carrying capacity

The relative reduction in density associated with a reduction in carrying capacity varied with the external recruitment level and within population spawning frequency (Fig. 2). Reducing carrying capacity under low (R = 20 larvae per $\hbox {m}^2$) and medium external recruitment (R = 200 larvae per $\hbox {m}^2$) has low impact on adult population density when within population spawning does not occur or occurs only every 5 years (Fig. 2A,B). Increasing carrying capacity from 25 to 100% maximum carrying capacity, with annual within population spawning under all three external recruitment levels, shifts the trend in adult population density from decreasing to increasing (Fig. 2C). In contrast, complex interplay between external recruitment and competition is illustrated when, under high external recruitment (R = 2000 larvae per $\hbox {m}^2$), a reduction in carrying capacity to 25% of maximum carrying capacity always results in a greater than 75% reduction in adult population density across all within population spawning frequencies, though this does not impact adult density trends over time.

**Figure 3:**
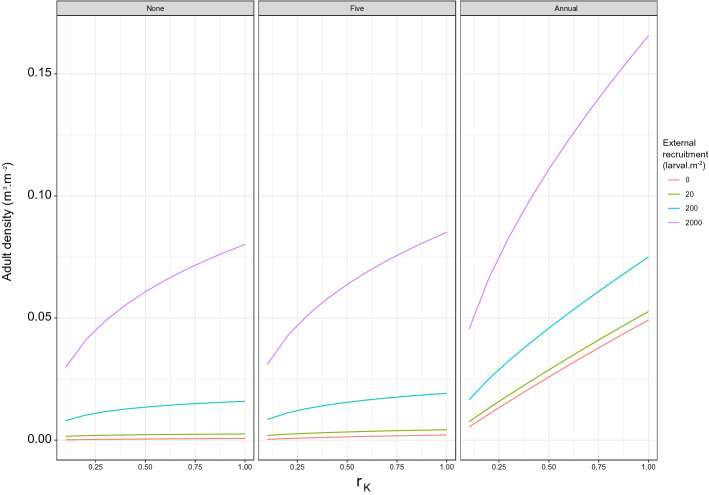
Averaged adult density of the wild population ($\hbox {m}^3\,\hbox {m}^{-2}$) at year 20 over $r_K$, the carrying capacity ratio, for 4 levels of external recruitment (orange—R = 0 $\hbox {larvae m}^{-2}$, green—R = 20 $\hbox {larvae m}^{-2}$, blue—R = 200 $\hbox {larvae m}^{-2}$, purple—R = 2000 $\hbox {larvae m}^{-2}$) with, from the left to the right, no spawning event (*None*), spawning events every 5 years (*Five* ), and annual spawning events (*Annual*). $r_K=1$ corresponds to the maximum carrying capacity and $r_K=0.5$ to 50% of the maximum carrying capacity.

Adult density under high external recruitment substantially exceeds density under any other combination of spawning frequency, recruitment level and carrying capacity (Fig. 3). Further, when external recruitment occurs under conditions of reduced carrying capacity, a reduction in adult density is seen due to the effect of reduced carrying capacity on adult growth. As a result adult density under medium external recruitment and maximum carrying capacity can exceed adult density under high external recruitment and reduced carrying capacity. The reduced carrying capacity limits the increase in adult density and therefore counterbalances the effect of external recruitment.

### Case study scenarios

#### Spawning patterns

**Figure 4:**
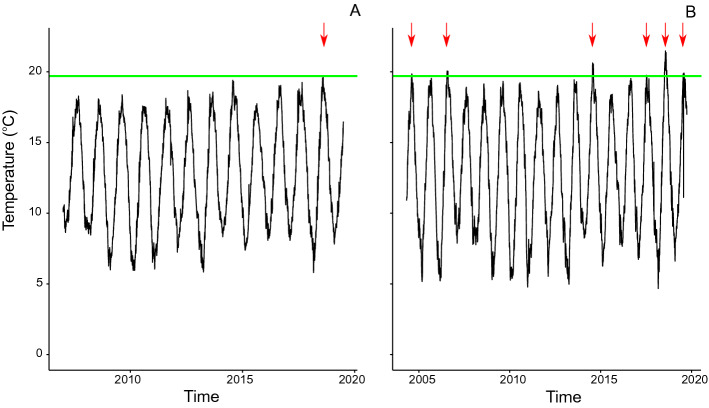
Daily average temperature ($^\circ \hbox {C}$) in Weymouth from 2006-01-01 to 2019-06-30 (**A**) and in Poole from 2004-05-01 to 2019-09-30 (**B**). The green horizontal line corresponds to the threshold temperature for spawning set at 19.7 $^\circ \hbox {C}$ and the red arrows to favorable conditions for spawning events.

According to temperature data collected in Weymouth from 2007-01-01 to 2019-06-30 (13 summers), the threshold temperature for spawning was met only once during this period, in summer 2018 (Fig. 4A). In Poole, the temperature data was collected from 2004-05-01 to 2019-09-30 (16 summers) during which the threshold temperature for spawning was exceeded 6 times (Fig. 4B). Under a 0.6 $^\circ \hbox {C}$ temperature increase scenario, the threshold temperature for spawning is reached 3 times in Weymouth and 12 times in Poole. These numbers increase to 11 in Weymouth and 16 in Poole (i.e. annually) under a 2 $^\circ \hbox {C}$ temperature increase scenario.

#### Differences between sites

**Figure 5:**
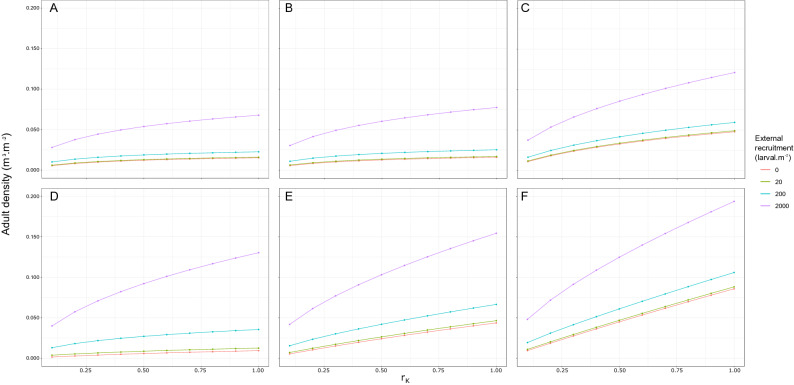
Averaged adult density of the wild population ($\hbox {m}^3\,\hbox {m}^{-2}$) at year 20 over $r_K$, the carrying capacity ratio, for 4 levels of external recruitment (orange—R = 0 $\hbox {larvae m}^{-2}$, green—R = 20 $\hbox {larvae m}^{-2}$, blue—R = 200 $\hbox {larvae m}^{-2}$, purple—R = 2000 $\hbox {larvae m}^{-2}$) at Weymouth under (**A**) observed temperature conditions, (**B**) a 0.6 $^\circ \hbox {C}$ temperature increase and (**C**) a 2 $^\circ \hbox {C}$ temperature increase, and at Poole under (**D**) observed temperature conditions, (**E**) a 0.6 $^\circ \hbox {C}$ temperature increase and (**C**) a 2 $^\circ \hbox {C}$ temperature increase.

Simulations indicate that under similar annual external recruitment and reduction in carrying capacity, adult density is higher in Poole than Weymouth (Fig. 5). In both locations, given similar carrying capacity, adults were at their lowest density in the absence of external recruitment and at their highest density under high external recruitment. The relative increase in adult density under low external recruitment compared to the absence of external recruitment is small and less than $0.012\,\hbox {m}^3\,\hbox {m}^{-2}$ across all scenarios. Conversely the relative increase in adult density under medium and high external recruitment is substantial, with adult density 3.8 and 14 times higher on average, respectively, compared to adult density in the absence of external recruitment and under maximum carrying capacity.

#### Changes in water temperature

Simulations reflecting increased water temperature in both Poole and Weymouth indicate an increase in adult density in both locations, under both temperature increase scenarios and all levels of external recruitment. In Weymouth, adult density did not substantially differ and remained below 75% of the maximum carrying capacity under observed conditions and the 0.6 $^\circ \hbox {C}$ temperature increase scenario (Fig. 5A,B). However, under the 2 $^\circ \hbox {C}$ temperature increase scenario, the adult density reaches 50% of the maximum carrying capacity under low and medium external recruitment, and exceeds maximum carrying capacity under high external recruitment (around $0.12\,\hbox {m}^3\,\hbox {m}^{-2}$ at year 20) (Fig. 5C). In Poole, under both temperature increase scenarios and under high external recruitment the adult density exceeds its carrying capacity (Fig. 5D,E,F). This is also observed under a 2 $^\circ \hbox {C}$ temperature increase scenario and medium external recruitment. It is worth noting that in Poole, under a 2 $^\circ \hbox {C}$ temperature increase scenario, in the absence of external recruitment, an already established population could reach a density close to carrying capacity (Fig. 5F).

The increase in temperature intensifies the effect of a reduction in carrying capacity under all external recruitment scenarios in both locations (Fig. 5). The reduction in carrying capacity is expected to induce a decrease in adult density, however the effect of reduced carrying capacity is more pronounced under temperature increase scenarios in Poole compared to Weymouth. Under high external recruitment, a 75% reduction in carrying capacity reduces adult density by 61% and 50% on average across all temperature scenarios in Poole and in Weymouth, respectively.

### Elasticity analysis

**Figure 6:**
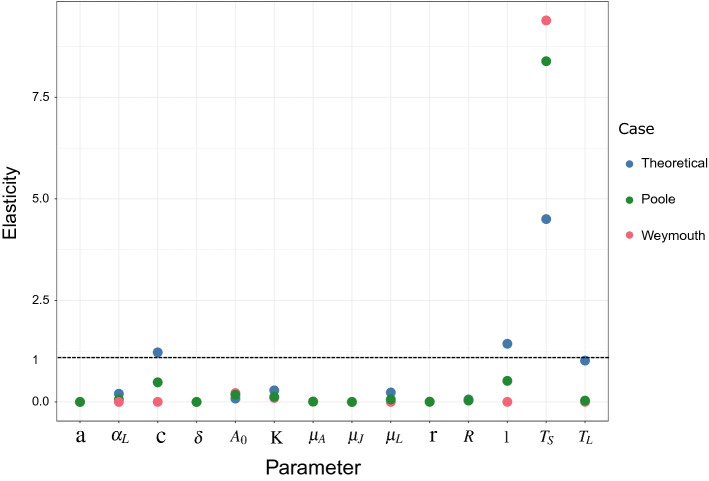
Elasticity analysis of model applied to the theoretical scenario (blue points) under favorable temperature conditions for annual spawning, and case studies Weymouth (pink points) and Poole (green points) under observed conditions for parameters defined in Table 1. The elasticity value reflects the proportional change in the adult oyster population density resulting from a 1% change in the parameter value and is dimensionless.

Elasticity analysis of mean adult density outputs across the 20 years time series from the theoretical model, under temperature conditions that allow annual spawning and in the absence of external recruitment, indicates high model sensitivity to spawning threshold temperature ($\xi _{T_S}=4.51$), number of larvae produced by adult volume ($\xi _{l}=1.43$) and conversion of juvenile into adult volume ($\xi _c=1.22$) (Fig. 6). Specifically, a 1% change in spawning threshold temperature, number of larvae produced by adult volume and conversion of juvenile into adult volume results in a 4.51% ($\xi _{T_S}=4.51$) and a 1.43% ($\xi _l=1.43$), and a 1.22% ($\xi _c=1.22$) change in adult population density respectively. Elasticity analysis applied to case study model parameter values, under observed temperature conditions and in the absence of external recruitment, also indicates high model sensitivity to spawning threshold temperature, with a 1% change in a spawning threshold temperature resulting in a 9.39% ( $\xi _{T_S}=9.39$) and 8.39% ( $\xi _{T_S}=8.39$) change in adult population density for Weymouth and Poole respectively. Sensitivity to other parameters is not evident with elasticities being strictly inferior to 1.

**Table 1:** Parameters for models (1)–(6).

Symbol	Description	Unit	Value	References
l	Number of larvae produced by $1\,\hbox {m}^3\,\hbox {m}^{-2}$ of adult oysters	number	20,000	^60^
$A_0$	Initial adult density	$\hbox {m}^3\,\hbox {m}^{-2}$	0.05	–
$\alpha _L$	Optimal larval development rate	$\hbox {d}^{-1}$	1/14	^14^
$T_S$	Minimal temperature for spawning	$^{\circ }\hbox {C}$	19.7	^11^
$T_L$	Minimal temperature for larval development	$^{\circ }\hbox {C}$	15	^14^
$T_J$	Minimal temperature for juvenile survival	$^{\circ }\hbox {C}$	3	^23^
$\mu _L$	Larval mortality rate	$\hbox {d}^{-1}$	1/14	^57^
$\delta$	Mortality rate due to the temperature	$\hbox {d}^{-1}$	0.99	^23^
$\mu _J$	Juvenile mortality rate	$\hbox {d}^{-1}$	$7.2.10^{-4}$	^57^
a	Daily rate of juvenile spat becoming adults	$\hbox {d}^{-1}$	1/365	^22^
c	Conversion rate of juveniles into adult volume	$\hbox {d}^{-1}$	$5.10^{-5}$	^57^
$\mu _A$	Adult mortality rate	$\hbox {d}^{-1}$	0.0015	^25^
r	Adult oyster growth rate	$\hbox {d}^{-1}$	0.001	^57^
K	Adult oyster carrying capacity	$\hbox {m}^3\,\hbox {m}^{-2}$	0.1	^11^
R	External recruitment level	number	0–2000	^60^

## Discussion

The Pacific oyster is one of the most important commercial shellfish worldwide but it can also be invasive outside its native range^5^. Widely introduced into Northern European waters in the mid-1960s to support the aquaculture industry at risk as a result of declining native oyster populations, spawning^3^ and external recruitment^34^ was thought to be inhibited by low water temperatures in the northmost regions. Persistence of wild oyster reefs is now heavily reported in North West Europe^10,12^, including on the South coast of England and Wales, and occasional isolated settlements in cooler Scottish waters^28,29^, which has been linked to recent warm summers that facilitate spawning^11,28,30–33^ and to external larval recruitment from local aquaculture sites and more distant populations, shipping, and live trade^34^. However, persistence of wild oysters can be unpredictable even under favorable temperature conditions^11,25^. To identify drivers of *M. gigas* population dynamics and persistence, this study explored the impact of increased spawning frequency reflecting future climate projections and its interaction with external larval recruitment and changes in carrying capacity, that may occur due to the partitioning of resources between wild and farmed Pacific oyster populations^38^. The study highlights that drivers of Pacific oyster population dynamics are complex and interacting, with key findings being: (1) in the absence of external recruitment wild populations can only increase in the long term and persist towards carrying capacity if they spawn annually, (2) in the absence of annual spawning, population extinction may be prevented by annual external recruitment, which needs to be substantial for the population to reach its carrying capacity, and (3) if sources of external recruitment compete with the wild population for resources the increase in density seen with external recruitment may be reduced, and in extreme cases reversed so that despite external recruitment population density is less that seen with no external recruitment.

Global climate change may have a substantial impact on the persistence and abundance of wild Pacific oyster populations by increasing spawning frequency, and there is a large literature describing the impact of warmer temperature on the presence of Pacific oyster in Northwestern European waters^10,12,25,26^. The present study provides further evidence that more frequent warm summers can facilitate persistence of wild oyster populations and increase the density of *M. gigas*. In our model, temperature directly dictates wild spawning occurrence and frequency, as well as larval and juvenile development and mortality. Low temperatures can limit the persistence of a wild population by inhibiting reproduction while high temperatures can intensify the frequency of spawning events and increase the growth rate of a wild population at a short time scale by increasing the larval recruitment. The sensitivity of the theoretical and case study model outputs to the spawning threshold temperature supports the idea that even a small temperature change may have a substantial impact on the Pacific oyster dynamics. Simulations indicate that wild populations may only experience a long-term increase in density and persist over a period of 20 years when temperatures facilitate spawning annually, implying that other factors are likely to be facilitating the long-term increase and persistence of Pacific oyster populations in UK waters. For example, while in our model reproduction patterns are linked to spawning temperature only, the gametogenesis process, initiated in winter and leading to sexual maturation prior to spawning in summer, is known to depend on both the temperature and the quantity of food availability^41^.

Larval recruitment from external sources under favorable environmental conditions has been identified as an important driver of the persistence and spread of *M. gigas* in Northern Europe^34^. The influence of external recruitment, including from Pacific oyster produced for aquaculture purposes, on wild population dynamics are however likely complicated. The literature explores both positive association between farm recruitment and oyster settlement^6,32^ and negative effect of competition for resource between farmed oysters and native filter feeders^35–38^ in close proximity. Results from this study suggests that the impact of annual external recruitment is dependent on resource competition and associated implications for the carrying capacity. Despite providing a source of external larval recruitment, it is possible that neighboring populations compete with a wild population for resources and reduce the density below that seen in populations receiving no external recruitment, through their impact on population carrying capacity and density dependent growth. However under favorable temperature conditions, where annual spawning occurs, simulations indicate that high external recruitment mitigates the effects of the reduced carrying capacity. This also suggests that in the absence of competition for resources, wild oyster populations can develop to their full carrying capacity and therefore reach higher densities than when in competition for resources.

Weymouth and Poole were selected as case study sites to provide further insight into the potential implications of temperature and external recruitment on Pacific oyster population dynamics and help validate the outputs of the theoretical model. Both locations have oyster farms and busy harbors and, given their location on the south coast of England where a number of wild populations are known to exist, may be at risk from external recruitment^42^. Despite this potential for external recruitment, no wild Pacific oyster populations have been observed in Weymouth^8^. Model simulations based on Weymouth temperature data indicate two possible reasons for this. Firstly, that lower temperatures in Weymouth result in too few spawning events to support the persistence of wild oysters and secondly, that recruitment from external sources, including the aquaculture site in close proximity, may be low and or in resource competition, thereby reducing the carrying capacity of the wild population, and its ability to persist. Further, the lack of wild populations in Weymouth suggests that temperature-independent external recruitment into the area is unlikely. In contrast, oyster reefs of low density (up to $0.003\,\hbox {m}^3\,\hbox {per m}^{2}$) have been reported in Poole^11^. Temperature records from Poole indicate potential for a greater number of spawning events in Poole compared to Weymouth. Model simulations based on Poole temperatures indicate that Pacific oysters can establish under any scenario, but that even in the presence of low to medium recruitment, resource competition may restrict the populations to low densities. However, the level of stocks in both farm sites is low (annual production of 10–200 tonnes), which suggests that competition is unlikely to occur between farmed and wild oyster populations but puts emphasis on the combined effect of temperature and external recruitment. Comparison of outputs from models based on the two case studies therefore supports the theory that the interaction between temperature, external recruitment and competition play a key role in determining oyster populations dynamics.

While the impact of temperature, external recruitment and competition was shown to be proportional to the frequency of spawning, level of recruitment, and resource partitioning, mechanisms influencing the persistence of *M. gigas* are difficult to assess. Therefore, predicting the success of management strategies to control persistent wild populations is challenging^43,44^ as many environmental, physiological, and biological factors may influence oyster population dynamics, including temperature^9,13,20^, habitat^45,46^, food^14,47^, salinity^48^, predation^32^, disease^49^ and potential for adaptive phenotypic plasticity under climate change^50^. In the absence of regular and robust routine monitoring, the model developed in this study provides a useful framework to help identify and evaluate potential control measures that could be applied to reduce wild oyster densities. For example, the model could be extended to incorporate and examine the merit of management approaches, such as manual removal of adult oysters, both in the short- and long-term, with changing environmental pressures^44^. The model also shows great robustness to parameter uncertainty and illustrates changes in wild oyster population density relative to interacting factors, providing a promising adaptable tool to predict the impact of underlying mechanisms facilitating population persistence.

Aquaculture impact on wild population persistence is undoubtedly complex and often controversial^33^. Oyster density in aquaculture sites can be optimized to maximize production based on resource availability^35,37,51^, which may induce a direct competition for nutrients^36,52^ and disturb reproduction^53^ of wild populations. In contrast, recent studies indicate that aquaculture sites may benefit wild oyster populations by diluting parasites through harvest, and thus reducing parasites load and disease in wild populations^54^. Given that many factors may be involved, with potentially complex interactions, further fieldwork and theoretical modeling will be valuable in understanding the effect of Pacific oyster farming on wild population dynamics. In particular, long-term monitoring of wild oyster abundance and reproductive patterns will provide essential knowledge to support and validate the model’s results, and shed light on potential temporal and spatial differences. Such insights may contribute to the much-needed evidence base required to make informed and balanced decisions with respect to policies relating to Pacific oyster aquaculture and wild population control.

## Methods

### Pacific oyster population model

**Figure 7:**
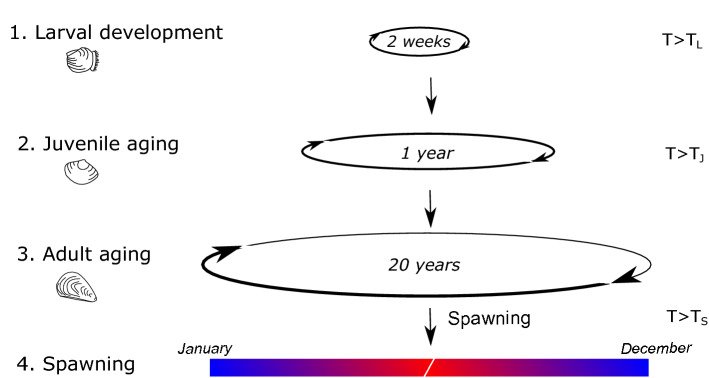
Oyster life-cycle: (1) Larvae are at a planktonic free-living stage, and develop and settle as juvenile spat above $T_L$ in a couple of weeks. (2) Juvenile spat are permanently attached to a substrate and develop into adult in 1–3 years. Juvenile spat cannot survive under $T_J$ and do not reproduce. (3) Adult oyster can live 20 years and up to 40 years and produce larvae. (4) In temperate regions, spawning occurs in warm months when the spawning temperature $T_S$ is reached.

Previously published differential equation-based models created to represent a stage-structured oyster population^39^ were adapted and expanded to describe larval, juvenile, and adult population dynamics under different spawning and external recruitment scenarios. Specifically, the model represents a discrete wild oyster population comprising 3 life-cycle stages: *L*, the free-swimming larval stage; *J*, the non-reproductive juvenile stage; and *A*, the reproductive adult stage (Fig. 7). Larvae and juvenile spat are expressed as number of free-living individuals per $\hbox {m}^2$ that do not occupy a specific volume. The adult population density is, however, expressed as volume per $\hbox {m}^2$ as once juvenile spat settle into adults they remain in place but continue to grow and occupy more volume, impacting the density-dependent rate of adult volume increase^39^. Expressing the adult population density as a volume is particularly relevant in habitat studies and allows resource and space dependent growth to be accounted for^40^.

Temperature is a key driver of oyster population dynamics with spawning, larval development and juvenile survival all temperature dependent. The Pacific oyster model developed in this study is semi-discrete^55^ allowing both continuous life traits, such as development and growth, and discrete temperature-dependent events, such as spawning (time $t=\tau _s$) and external recruitment (time $t=\tau _r$), to be linked to seasonal variability. The density of adult oysters is assumed to follow a logistic growth curve, limited by resources and space. Adult mortality is modeled by using a linear mortality relationship^56^. Juvenile spat fuel the adult population and are directly impacted by the number of larvae in the population^57^. The model thus reads:

1 $$\begin{aligned} t \ne \{\tau _s,\tau _r\} \left\{ \begin{array}{lcl} \dot{L}&{}=&{}-\alpha _{L}L(f_{L1}(T_p)+f_{L2}(T_p))-\mu _{L}L,\\ \dot{J}&{}=&{}\alpha _{L}Lf_{L1}(T_p)-\delta _{J}Jf_{J}(T_p)-\mu _{J}J-aJ,\\ \dot{A}&{}=&{}acJ+\displaystyle rA\left( 1-\frac{A}{K}\right) - \mu _{A}A,\\ \end{array} \right. \end{aligned}$$

with $T_p$ the temperature, $\alpha _L$ the optimal larval development rate at temperature $T_p=T_S$, $\mu _{L}$, $\mu _{J}$ and $\mu _{A}$ the mortality rates encompassing predation, disease and natural mortality for larvae, juvenile spat and adult oysters, respectively. $\delta$ is the juvenile mortality rate at temperature $T_p=T_J$. *a* is the daily rate of juvenile spat becoming adults, and *c* the conversion rate of a juvenile spat into adult volume. *r* is the adult volume growth rate, and *K* the population carrying capacity for adult oysters.

#### Temperature-dependent stage development

$f_{L1}(T_p)$, $f_{L2}(T_p)$ and $f_{J}(T_p)$ reflect the linear temperature-development rate for larvae, the exponential temperature-mortality rate for larvae and the exponential temperature-mortality rate for juveniles, respectively, and depend on $T_L$ the minimal temperature for larval development and $T_J$ the minimal temperature for juvenile survival. These functions are expressed as:

2 $$\begin{aligned}&T_p>T_L \left\{ \begin{array}{lcl} f_{L1}(T_p)&{}=&{}T_p \displaystyle \frac{T_p-T_L}{T_S-T_L},\\ f_{L2}(T_p)&{}=&{}0,\\ f_{J}(T_p)&{}=&{}0.\\ \end{array} \right. \end{aligned}$$

3 $$\begin{aligned}&T_J<T_p<T_L \left\{ \begin{array}{lcl} f_{L1}(T_p)&{}=&{}0,\\ f_{L2}(T_p)&{}=&{}\displaystyle \frac{e^{T_L}}{e^{T_p}},\\ f_{J}(T_p)&{}=&{}0.\\ \end{array} \right. \end{aligned}$$

4 $$\begin{aligned}&T_p<T_J \left\{ \begin{array}{lcl} f_{L1}(T_p)&{}=&{}0,\\ f_{L2}(T_p)&{}=&{}0,\\ f_{J}(T_p)&{}=&{}\displaystyle \frac{e^{T_J}}{e^{T_p}}.\\ \end{array} \right. \end{aligned}$$

#### Temperature-dependent spawning

Within a population, spawning is assumed to occur as a single event when the seawater temperature reaches $T_S$, the minimal temperature for spawning, for the first time in the calendar year ($t=\tau _s$). This is modeled as a discrete number of larvae produced by volume of oysters and reads:

5 $$\begin{aligned} t=\tau _s \left\{ \begin{array}{lcl} L_{\tau }&{}=&{}lA_{(\tau -1)}+L_{(\tau -1)}\\ J_{\tau }&{}=&{}J_{(\tau -1)}\\ A_{\tau }&{}=&{}A_{(\tau -1)}\\ \end{array} \right. \end{aligned}$$

The oyster sex ratio, known to vary markedly with age and environmental conditions^58^, is not considered here. *l* is therefore the average number of larvae produced by total oyster volume per $\hbox {m}^2$.

#### External recruitment

Annual external larval recruitment can occur as a result of human mediated pathways such as shipping and live trade, or originate from oyster farms, or other long distant wild populations that can spawn annually because they are under more favorable environmental conditions compared to the simulated wild oyster population^34^. More favorable conditions are particularly experienced by farmed oysters produced in shallow and sheltered harbours^11^.

External recruitment is modeled as a single annual discrete event ($t=\tau _r$) and reads:

6 $$\begin{aligned} t=\tau _r \left\{ \begin{array}{lcl} L_{\tau _r}&{}=&{}R+L_{(\tau _r-1)},\\ J_{\tau _r}&{}=&{}J_{(\tau _r-1)},\\ A_{\tau _r}&{}=&{}A_{(\tau _r-1)},\\ \end{array} \right. \end{aligned}$$

with *R* the number of larvae recruited into the population per $\hbox {m}^2$ during the event. If within population spawning occurs simultaneously with external recruitment, the total number of recruited larvae per $\hbox {m}^2$ becomes the combination of models (5) and (6).

#### Carrying capacity

Local Pacific oyster farms can compete for resources with wild Pacific oyster populations^35–37^. Therefore, it is assumed that the carrying capacity of the modeled population is dependent on the partitioning of resources between it and a farm, and is expressed as a proportion $r_K$ of the maximum carrying capacity *K*. *K* impacts the density-dependent adult growth directly, so that any change in the carrying capacity impacts the adult population density directly. Adult density is also directly influenced by the conversion rate of juvenile number into adult volume and by the number of juveniles. Carrying capacity may therefore be exceeded when larval recruitment is high.

### Theoretical scenario

A time-series of annual water temperature was simulated over 20 years using a basic cosine wave $T_p(t)=A_pcos(wt-\phi )+T_M$ with *A*_*p*_, the amplitude or peak deviation of the function from zero (set at $5^{\circ }C$), *w*, the rate of change of the function argument (set at 0.01721421), $\phi$, the phase (set at 60 days), $T_M$ the average temperature (set at 13 $^\circ$C), and *t*, the time as day^59^. Temperature was assumed to exhibit the same pattern each year and to never reach $T_S$ (maximum temperature was $18^{\circ }C$). Annual water temperatures were increased by 2$^{\circ }C$ to ensure that $T_S$ was reached at different frequencies, mimicking climate change and reflecting 6 spawning scenarios: (1) no event, (2) once at year 10, (3) once at year 5 and once at year 15, (4) every 5 years, (5) biannually or (6) annually.

The modeled population was simulated under the 6 spawning frequency scenarios with no external recruitment (Fig. 1). The modeled population was also simulated under 3 different recruitment levels^60^ ($R={20;{ }200;{ }2000}$ larvae per $\hbox {m}^{2}$), reflecting the potential range of external recruitment levels from very low level external recruitment, indicative of long-distance dispersion to high level external recruitment from a farm or wild population in close proximity. The combined effect of spawning frequency and external recruitment was then examined by simulating the modeled population under each recruitment level, combined with spawning scenarios 1), 4) and 6), with the effect of farm competition examined through alteration of the carrying capacity proportion. Average adult densities at year 20 and time-series dynamics of the adult density are reported.

### Case study: Weymouth and Poole

**Figure 8:**
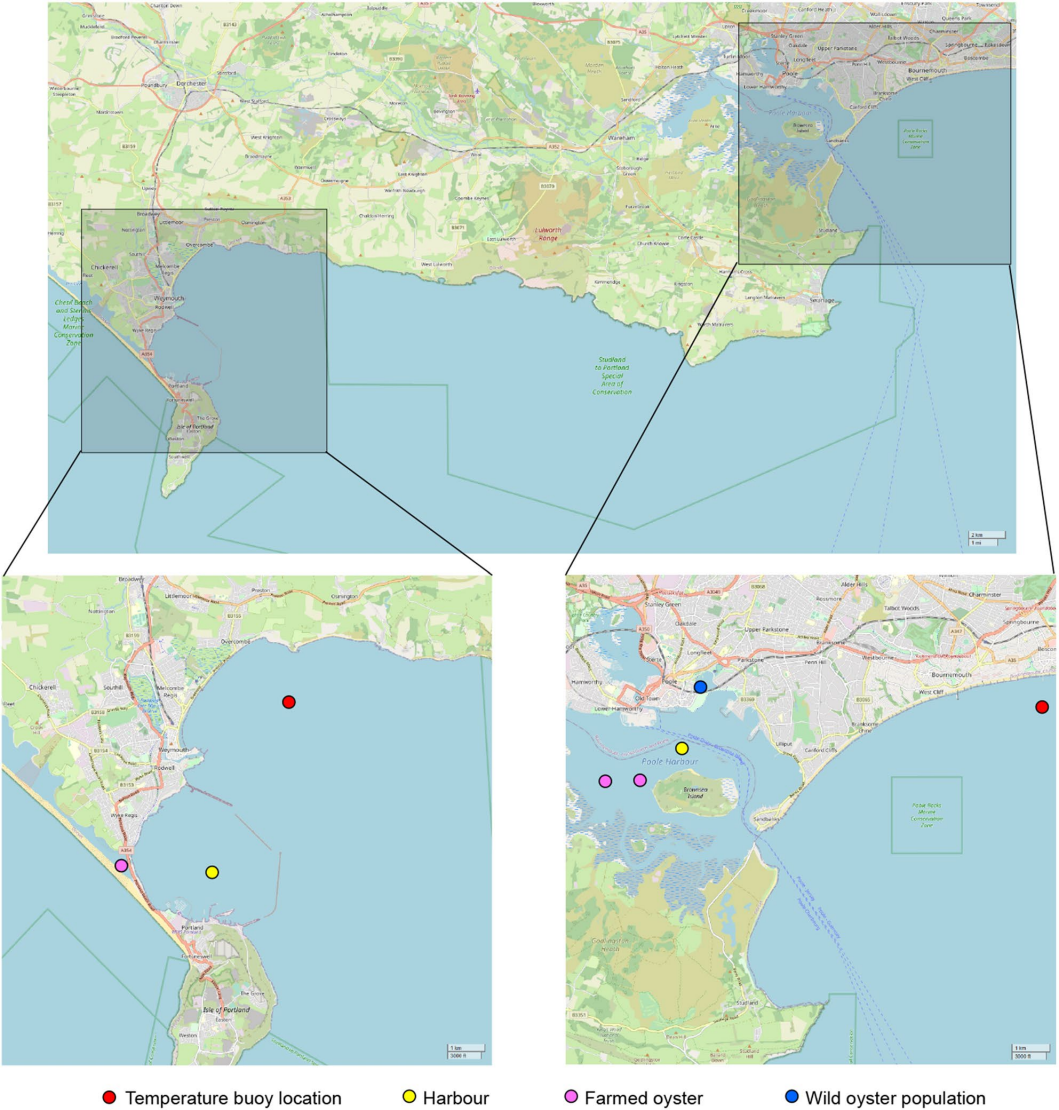
Location of Weymouth (left) and Poole (right) with temperature buoy location (red circle), harbor (yellow circle), farmed oyster (purple circle) and established wild oyster population (blue circle), created using Inkscape 0.91^69^ and OpenStreetMap (copyright OpenStreetMap contributors, licensed under the Open Data Commons Open Database License (ODbL) by the OpenStreetMap Foundation (OSMF), cartography licensed as CC BY-SA).

Weymouth and Poole are two towns, situated on the south coast of England, 15 miles apart as the crow flies (Fig. 8). Weymouth is in close proximity to oyster farms (Portland Harbour and the Fleet), but despite Pacific oysters being farmed since 1988 no wild settlement has been reported despite favorable habitat for settlement being abundant^8^. Wild *M. gigas* are present in Poole Harbour at a very low density ranging from $8.3.10^{-4}$ to 3 individuals per $\hbox {m}^{2}$^11^. Farming Pacific oysters is a long-standing tradition in Poole, initiated with the introduction of *M. gigas* in 1890^3^. Temperature time-series for each location was obtained from the National Network of Regional Coastal Monitoring Programmes of England^61^ (www.channelcoast.org) and detailed temperatures continuously recorded and averaged over 30 minutes from 2007-01-01 to 2019-06-30 in Weymouth and from 2004-05-01 to 2019-09-30 in Poole. A daily mean for each location was calculated and used in this study.

For each location, the presence of an already established wild oyster population is assumed and modeled under three temperature scenarios, observed temperatures, observed temperatures increased by 0.6 $^\circ \hbox {C}$ (representative of the lowest climate change prediction, RCP of 2.6 by 2050), and observed temperatures increased by 2 $^\circ \hbox {C}$ (representative of the highest climate change prediction, RCP of 8.5 by 2050) alone^62^, and in combination with three different external recruitment levels ($R={20; 200; 2000}$ larvae per $\hbox {m}^{2}$) and varied carrying capacity proportions.

### Parameterization and elasticity analysis

Parameterization of the model was based on evidence found in key literature (Table 1). When necessary, assumptions were made. Specifically, the optimal larval development rate was assumed to be $1/14 \,\mathrm{d}^{-1}$ based on the 2-week larval development duration under optimal temperature conditions^14,15,19,20^. In addition, the minimal temperature for spawning ($T_S=19.7\,{}^\circ$C) was estimated from experimental work aiming to determine the annual reproductive cycle and reproductive patterns of adult Pacific oysters in relation to temperature conditions in Poole Harbour^11,63^.

Due to the model parameter uncertainty and variability observed in Pacific oyster life-history traits elasticity analysis was performed to help understand how changes in parameters affect model outputs (adult oyster population density)^64^. The elasticity can be interpreted as a proportional sensitivity, i.e. a proportional change in the population size resulting from a proportional change in the parameter. The elasticity is therefore dimensionless and independent of the parameter scale, which allows comparison between parameters. Since adult oyster population density does not always reach a stable equilibrium but can oscillate in the long term, a mean adult density $A_m$ across the time series was calculated for each parameter simulation. Specifically, model elasticity was performed by calculating the proportional change in $A_m$ that resulted from a proportional change in a parameter *p* value within its range, expressed as partial differential equations^65,66^:

$$\begin{aligned} \xi _p=\displaystyle \frac{p}{A_m}\frac{\partial A_m}{\partial p}. \end{aligned}$$

The elasticity analysis was applied to the theoretical case with annual spawning events, and to the Weymouth and Poole case studies under observed temperature conditions in the absence of external recruitment.

Data analyses and modeling simulations were all conducted using the statistical software R^67^ and the deSolve package^68^.
